# Long-term prognostic value of LDL-C, HDL-C, lp(a) and TG levels on cardiovascular disease incidence, by body weight status, dietary habits and lipid-lowering treatment: the ATTICA epidemiological cohort study (2002–2012)

**DOI:** 10.1186/s12944-022-01747-2

**Published:** 2022-12-19

**Authors:** Michael Georgoulis, Christina Chrysohoou, Ekavi Georgousopoulou, Evangelia Damigou, Ioannis Skoumas, Christos Pitsavos, Demosthenes Panagiotakos

**Affiliations:** 1 Department of Nutrition and Dietetics, School of Health Sciences and Education, Harokopio University of Athens, 17676 Athens, Greece; 2 First Department of Cardiology, School of Medicine, National and Kapodistrian University of Athens, Athens, Greece

**Keywords:** Lipidemic profile, Low-density lipoprotein cholesterol, Lipoprotein(a), High-density lipoprotein cholesterol, Hyperlipidemia, Dyslipidemia, Triglycerides, Cardiovascular risk

## Abstract

**Background:**

The link between blood lipids and cardiovascular disease (CVD) is complex. Our aim was to assess the differential effect of blood lipids on CVD risk according to age, sex, body weight, diet quality, use of lipid-lowering drugs and presence of hypercholesterolemia.

**Methods:**

In this secondary analysis of the ATTICA prospective cohort study, serum blood lipids, i.e., total cholesterol (TC), low-density lipoprotein cholesterol (LDL-C), high-density lipoprotein cholesterol (HDL-C), triglycerides (TG) and liproprotein(a) [Lp(a)], and sociodemographic, anthropometric, lifestyle and clinical parameters were evaluated at baseline (2001/2002) in 2020 CVD-free men and women. CVD incidence was recorded at the 10-year follow-up (2011/2012).

**Results:**

All blood lipids assessed were univariately related to CVD risk; however, associations remained significant only for HDL-C and TG in multivariate models adjusted for age, sex, body mass index, smoking, Mediterranean Diet Score, physical activity, presence of hypercholesterolemia, hypertension and diabetes mellitus, use of lipid-lowering drugs, and family history of CVD [RR per 1 mg/dL (95% CI): 0.983 (0.967, 1.000) and 1.002 (1.001, 1.003), respectively]. In stratified analyses, TC and LDL-C predicted CVD risk in younger subjects, normal-weight subjects, and those not on lipid-lowering drugs, while HDL-C and TG were significant predictors in older subjects, those with low adherence to the Mediterranean diet, and hypercholesterolemic subjects; a significant effect on CVD risk was also observed for TG in males, overweight participants and lipid-lowering medication users and for Lp(a) in older subjects and females (all *p* ≤ 0.050).

**Conclusions:**

The impact of blood lipids on CVD risk differs according to several biological, lifestyle and clinical parameters.

## Background

Dyslipidemia, defined as any abnormal alteration in the plasma lipid profile, represents a significant modifiable risk factor for the development of cardiovascular disease (CVD). The global prevalence of dyslipidemia has been estimated to be as high as 40%, and blood lipid abnormalities account for more than one-third of deaths caused by ischemic heart disease or ischemic stroke in both the developed and developing world [1]. Among the various blood lipid indices, total cholesterol (TC) and low-density lipoprotein cholesterol (LDL-C) have traditionally stood out in relation to CVD risk. On the one hand, TC is currently utilized as the initial screening lipidemic index in most total CVD risk estimation systems/algorithms, such as the Systematic Coronary Risk Estimation (SCORE) of the European Society of Cariology, along with age, sex, systolic blood pressure and smoking status [2]. On the other hand, accumulated research has highlighted the retention of apo-B-containing lipoproteins, most importantly LDL-C but also very-low density lipoproteins and remnant chylomicrons, within the arterial wall as the key initiating event in atherogenesis; therefore, LDL-C is currently the primary target for lifestyle and pharmacological interventions to prevent CVD with specific goals relevant to the TC-based calculated total CVD risk [3].

Despite the significant role of TC and LDL-C in CVD screening, prevention and follow-up, a high residual CVD risk has been reported in statin-treated patients [4–6]; this observation suggests that focusing solely on TC or LDL-C might result in underestimating CVD risk in a considerable proportion of the population and highlights the need for investigating the prognostic ability of other lipid/lipoprotein metabolism biomarkers on CVD risk, even in individuals with adequate control of hypercholesterolemia. Indeed, research has shown that a lipidemic profile characterized by low high-density lipoprotein cholesterol (HDL-C), low apolipoprotein A1 (the major protein component of HDL particles), high triglycerides (TG) and a high ratio of TC/LDL-C:HDL-C can better predict CVD risk in both the general CVD-free population and statin-treated coronary patients compared to a lipidemic profile characterized by high TC and high LDL-C [7–9]. Moreover, lipoprotein(a) [Lp(a)], a plasma lipoprotein consisting of an LDL-like particle linked to apolipoprotein(a), is a novel and highly discussed CVD biomarker, given its strong prothrombotic effects because of the structural similarity of apolipoprotein(a) with antifibrinolytic factors, its ability to stimulate the production of proinflammatory cytokines, and its detrimental effect on the pathophysiology of atherosclerosis due to the accumulation of Lp(a) cholesterol within the arteries [10, 11].

Despite the abundance of available data on the link between various blood lipids and CVD risk, their differential association in subgroups of the general population remains poorly studied. As previously stated, the identification of appropriate lipid biomarkers is crucial not only in untreated apparently healthy individuals to assess total CVD risk but also in those under lipid-lowering medication to assess residual CVD risk and optimize health outcomes [12]. Other traditional CVD risk factors might also be of relevance when testing the association between lipidemic profile and CVD risk. For example, age and sex differences in CVD epidemiology and burden exist, and the importance of heart disease is increasingly recognized in younger adults and women; however, age- and sex-stratified associations between blood lipids and CVD risk have not been extensively studied [13, 14]. Moreover, body weight and diet quality can influence CVD risk, a secondary effect to their impact on CVD risk factors, including blood lipids [15, 16]. In this context, several epidemiological and clinical studies have revealed that a Mediterranean-style diet can lead to a substantial CVD risk reduction compared to a Western-type diet [17, 18]; however, it remains unclear whether the level of adherence to the Mediterranean diet can influence the relationship between blood lipids and CVD.

Given the aforementioned, the traditional simplistic view of the link between the lipidemic profile and CVD is gradually changing in light of novel data, and more research is required to identify blood lipid biomarkers that can optimally assess CVD risk and serve as endpoints for CVD prevention, as well as to understand how these biomarkers are differentially related to CVD risk based on other biological, clinical and lifestyle CVD risk factors. To this end, we aimed to explore and compare the predictive ability of TC, LDL-C, HDL-C, TG, and Lp(a) on 10-year CVD risk in a large, adult, community-based, free-living population who participated in the ATTICA prospective epidemiological study (2002–2012). Our secondary aim was to explore whether the link between blood lipid biomarkers and CVD risk differentiates according to other important biological, clinical and lifestyle characteristics, namely, age, sex, body weight status, quality of diet (using the level of adherence to the Mediterranean diet as an indicator), use of lipid-lowering medication and presence of hypercholesterolemia.

## Methods

### Study design

The ATTICA study is a prospective, observational, cohort investigation designed to identify sociodemographic, lifestyle, biochemical and clinical risk factors for the development of CVD among non-institutionalized, apparently healthy, adult males and females living in the capital province of Greece. The study protocol can be found in detail elsewhere [19].

### Bioethics

The study was conducted in accordance with the Declaration of Helsinki [20], and approval was obtained from the Ethics Committee of the 1st Department of Cardiology of the National and Kapodistrian University of Athens (code: 017/01.05.2001, date: May 1, 2001).

### Sample

The baseline phase of the ATTICA study (2001–2002) was conducted in the greater metropolitan area of Athens (78% urban municipalities). The sampling was random, based on the characteristics of the study population (distribution of age and sex), and performed in 27 different cities of the Attica region to account for spatial heterogeneity. A total of 4056 individuals were initially invited, of which 3042 (75% participation rate) provided written informed consent and were enrolled (1514 males aged 46 ± 13 years and 1528 females aged 45 ± 13 years). Upon enrollment, participants’ medical, biochemical, anthropometric, lifestyle and sociodemographic characteristics were assessed by trained health professionals, including general practitioners, nurses, cardiologists and dietitians, based on standardized procedures as described below [19]. Participants were followed-up for 10 years, and the association between selected baseline blood lipids and the 10-year CVD incidence (the main endpoint of the study) was explored after stratification for biological, lifestyle and clinical covariates.

### Baseline assessment

#### Lipidemic profile indices

Morning 12-hour fasting blood samples were collected from an antecubital vein, and serum was extracted and frozen at − 80 °C until analysis. Blood lipid biomarkers, i.e., TC, HDL-C, TG and Lp(a), were measured in a certified laboratory using appropriate biochemical assays as previously described [8]. Internal quality control was applied to blood lipid measurements, based on which the intra- and interassay coefficients of variation of TC, TG, HDL-C and Lp(a) did not exceed 9, 4, 4 and 4%, respectively. The Friedewald formula was utilized to calculate LDL-C based on TC, HDL-C and TG values [21]. All lipid biomarkers were also categorized as normal or abnormal based on cutoff values proposed in the latest European guidelines for dyslipidemias [22], i.e., 200 mg/dL for TC, 100 mg/dL for LDL-C, 40 mg/dL in males and 50 mg/dL in females for HDL-C, 150 mg/dL for TG and 50 mg/dL for Lp(a).

#### Medical, sociodemographic and clinical characteristics

Participants’ medical status was evaluated through face-to-face interviews and included a history of risk factors for CVD, i.e., hypertension, hypercholesterolemia and diabetes mellitus, and the use of relevant medication, such as lipid-lowering, antihypertensive and antidiabetic drugs. A sphygmomanometer was used by a trained cardiologist to measure blood pressure, with the subject sitting and after a 30-minute rest; a total of three measurements were recorded, and their average was used for analyses. Participants with systolic blood pressure ≥ 140 mmHg, diastolic blood pressure ≥ 90 mmHg, and those under blood pressure-lowering medication were classified as hypertensive. Demographic data (age, sex and marital status) and socioeconomic characteristics (annual income, years of education and type of occupation) were also recorded as previously described [19]. Four categories of financial status (≤8000 €, medium: 8001–10,000 €, high: 10,001–20,000 € and very high: > 20,000 €) and three categories of educational level (low: 0–6 years, medium: 7–13 years, and high: ≥14 years) were extracted based on annual income and total years of primary, secondary and tertiary education, respectively.

#### Anthropometric and lifestyle parameters

Height (m) and body weight (kg) were measured following a standardized protocol. Body mass index (BMI) was calculated as weight:height^2^, and participants were classified as overweight if BMI was 25.0–29.9 kg/m^2^ and obese if BMI was ≥30.0 kg/m^2^. Waist circumference (WC) was measured in cm using a nonelastic tape; the measurement was performed in a standing position, at the midpoint between the iliac crest and lowest rib, and at the end of normal expiration [23]. WC values ≥102 cm for men and ≥ 88 cm for women were considered elevated based on international criteria [24]. The short-form International Physical Activity Questionnaire was used to evaluate physical activity level [25]; participants were categorized as physically active if they reported performing any kind of physical activity of specific intensity and duration for > 1 day/week; otherwise, they were considered sedentary. Smoking habits were also evaluated, and subjects were classified as current smokers (≥1 cigarette/day), former smokers (smoking cessation for ≥12 months prior to baseline evaluation), and never smokers. To evaluate total exposure to tobacco, the duration of smoking (in years) was multiplied by the number of cigarette packs consumed per day (considering that each pack contains 20 cigarettes), and pack-years were calculated for each participant.

A validated 156-item semiquantitative food-frequency questionnaire [26] was used to evaluate participants’ habitual diet. Based on the consumption of foods, beverages and food groups, the a-priori Mediterranean Diet Score (MedDietScore) index [27] was calculated to evaluate participants’ level of adherence to the Mediterranean diet. In more detail, the MedDietScore incorporates the consumption of 11 dietary components, i.e., full-fat dairy products, fruits, vegetables, whole grains, potatoes, legumes, poultry, fish, red meat, olive oil and alcohol, which are scored on a 0–5 scale (for Mediterranean foods/food groups, this scale ranges from 0 – very rare consumption to 5 – very frequent consumption; the opposite scoring is applied to foods/food groups that are not considered part of the Mediterranean diet; for alcohol, consumption of 0 and > 7 servings per day is given a score of 0, and the consumption of 6–7, 5–6, 4–5, 3–4 and < 3 servings per day is given a score of 1 to 5, respectively, assuming 12 g of ethanol in a typical serving). The MedDietScore index obtains a total score of 0–55, with increasing values indicating a higher proximity to the Mediterranean diet. Based on the median MedDietScore value of the study population, participants were further classified into two groups: those exhibiting low adherence (below the median, i.e., < 26.5) and those exhibiting high adherence to the Mediterranean diet (equal to or above the median, i.e., ≥26.5).

### Follow-up assessment

Participants were followed-up at 10 years after enrollment (2011/2012). They were initially contacted by telephone and then evaluated through face-to-face interviews by the research team. Of the 3042 subjects who participated in the baseline phase of the ATTICA study, 2583 were re-evaluated (85% participation rate), and 459 were lost to follow-up (224 were not found due to missing or erroneous contact information provided at baseline, and 235 declined to participate in the 10-year re-evaluation). The follow-up assessment included information on participants’ vital status (death from any cause) and the development of nonfatal and fatal CVD, i.e., myocardial infarction, stroke, angina pectoris, congestive heart failure (congestive heart disease and right ventricular failure secondary to left heart failure) and left ventricular failure (cardiac asthma, left heart failure, edema of lung and pulmonary edema), other identified forms of ischemia, and chronic arrhythmias, which were retrieved from participants’ medical records. Accurate 10-year CVD incidence data were obtained for 2020 participants (66% of the study population), who comprised the final analyzed sample.

### Statistical analyses

All analyses were performed in STATA software version 15 (StataCorp. 2017. College Station, TX, USA). Statistical significance was set at 0.050. Data are presented as relative frequencies for categorical variables, mean ± standard deviation for normally distributed continuous variables and median (1st, 3rd quartile) for skewed continuous variables. Normality was evaluated through the Kolmogorov–Smirnov test. Differences between participants who experienced a CVD event throughout the study period and their CVD-free counterparts were tested through Pearson’s chi-square test for categorical variables, Student’s t test for normally distributed numerical variables (Levene’s test was performed prior to Student’s t test to explore the homogeneity of variance) and the Mann–Whitney test for skewed numerical variables. The incidence of CVD was estimated as the number of new CVD cases divided by the number of subjects re-evaluated at 10 years. Multivariate Cox regression models were utilized to extract the relative risks (RRs) and 95% confidence intervals (95% CIs) of developing a CVD event throughout the 10-year study period in relation to baseline TC, LDL-C, HDL-C, TG, and Lp(a) levels. Blood lipid biomarkers were inserted into the models both as continuous variables and as categorical ones indicating abnormal levels. Confounders included participants’ age, sex, BMI, smoking status, MedDietScore, physical activity level, presence of hypertension, hypercholesterolemia and diabetes mellitus at baseline, use of lipid-lowering medication, and family history of CVD. Models’ goodness of fit, classification ability and predictive power were evaluated through the Negelkerke adjusted R-square (*R*^*2*^) and Harrell’s C of inverse RR (the proportion of all subject pairs with concordant predictions and outcomes). The percentage (%) of improvement in the models’ predictive ability after the introduction of each blood lipid biomarker was also assessed through the continuous net reclassification improvement (cNRI). Sensitivity analyses were stratified by age (≤45-year-olds vs. > 45-year-olds), sex (males vs. females), body weight status (normal weight vs. overweight/obese), level of adherence to the Mediterranean diet (high vs. low), use of lipid-lowering medication (yes vs. no), and presence of hypercholesterolemia (TC ≥200 mg/dL vs. TC < 200 mg/dL).

## Results

During the 10-year follow-up period, 317 subjects (198 males and 119 females) experienced a CVD event, leading to a crude CVD incidence of 15.7% (males: 19.7%, females: 11.7%) and an annual CVD incidence of 146 cases per 10,000 subjects (males: 182 per 10,000, females: 110 per 10,000). The baseline characteristics of the study population according to 10-year CVD incidence are shown in Table 1. Compared to CVD-free participants, those who experienced a CVD event throughout 10 years were older, most frequently male, had a lower educational level, higher BMI and WC, higher overweight/obesity and abdominal adiposity rates, reported a lower level of adherence to the Mediterranean diet and a greater smoking burden assessed through pack-years, and had a higher prevalence of hypertension and diabetes mellitus at baseline (all *P* < 0.001). Subjects who experienced a CVD event during the study period also exhibited a more detrimental lipidemic profile than CVD-free subjects, including higher TC (*P* < 0.001), LDL-C (*P* < 0.001), TG (*P* < 0.001) and Lp(a) (*P* = 0.052), lower HDL-C (*P* < 0.001), and a higher prevalence of abnormal TC, LDL-C, HDL-C and TG levels (all *P* < 0.001).

**Table 1:** Sociodemographic, lifestyle, biochemical and clinical characteristics of the study population according to 10-year CVD incidence (*n* = 2020)

	CVD-free (***n*** = 1703)	CVD (***n*** = 317)	***P*** value^**a**^
Age, years	42.9 ± 12.8	57.8 ± 13.2	< 0.001
Males, %	47.4	62.5	< 0.001
Educational level, %			< 0.001
low	15.0	37.5	
medium	46.3	39.4	
high	38.7	23.0	
Financial status, %			0.240
low	22.3	24.2	
medium	32.2	29.9	
high	32.3	37.6	
very high	13.2	8.3	
BMI, kg/m^2^	26.0 ± 4.5	27.9 ± 4.5	< 0.001
25–30 kg/m^2^, %	38.8	47.6	< 0.001
≥ 30 kg/m^2^, %	16.4	27.1	< 0.001
WC, cm	89.3 ± 15.1	97.3 ± 13.7	< 0.001
Increased WC, %	51.1	67.8	< 0.001
Current smokers, %	41.9	35.3	0.028
Ever smokers, %	54.5	56.8	0.459
Pack-years	320 (140, 600)	600 (260, 1000)	< 0.001
MedDietScore (0–55)	26.4 ± 6.2	22.8 ± 6.5	< 0.001
MedDietScore < 26.5, %	55.5	88.3	< 0.001
Physically active, %	40.9	40.7	0.935
TC, mg/dL	190 (163, 218)	203 (178, 232)	< 0.001
TC ≥200 mg/dL, %	39.3	53.5	< 0.001
LDL-C, mg/dL	119 (95, 144)	125 (107, 155)	< 0.001
LDL-C ≥ 100 mg/dL, %	70.3	83.2	< 0.001
HDL-C, mg/dL	48 (39, 57)	43 (36, 53)	< 0.001
HDL-C < 40/50 mg/dL, %	41.2	55.4	< 0.001
TG, mg/dL	94 (66, 137)	129 (93, 182)	< 0.001
TG ≥150 mg/dL, %	20.7	39.0	< 0.001
Lp(a), mg/dL	10.9 (4.6, 23.5)	13.0 (5.0, 27.4)	0.052
Lp(a) ≥50 mg/dL, %	8.2	11.3	0.079
Use of lipid-lowering meds, %	8.1	26.5	< 0.001
Hypertension, %	28.0	50.7	< 0.001
Diabetes mellitus, %	4.6	21.5	< 0.001

Categorical variables are presented as relative frequencies, while numerical variables are presented as the mean ± standard deviation or median (1st, 3rd quartile) if normally distributed or skewed, respectively

^a^*P* values for between-group comparisons, as derived from Pearson’s chi-square test for categorical variables, Student’s t test for normally distributed numerical variables and the Mann–Whitney U test for skewed numerical variables

*BMI* Body mass index, *CVD* Cardiovascular disease, *HDL-C* High-density lipoprotein cholesterol, *LDL-C* Low-density lipoprotein cholesterol, *Lp(a)* Lipoprotein(a), *MedDietScore* Mediterranean Diet Score, *TC* Total cholesterol, *TG* Triglycerides, *WC* Waist circumference

In univariate analysis, all lipid biomarkers assessed were significantly associated with CVD risk. A 10-unit (mg/dL) increase in TC, LDL-C, TG and Lp(a) was associated with a 7% (RR per 1 mg/dL: 1.007, 95% CI: 1.002, 1.010), 6% (RR per 1 mg/dL: 1.006, 95% CI: 1.003, 1.010), 3% (RR per 1 mg/dL: 1.003, 95% CI: 1.002, 1.003) and 7% (RR per 1 mg/dL: 1.007, 95% CI: 1.002, 1.012) increased 10-year risk of CVD, respectively. In contrast, CVD risk decreased by 2.2% for every unit (mg/dL) increase in HDL-C (RR per 1 mg/dL: 0.978, 95% CI: 0.968, 0.989). Similarly, the univariate association between the presence of abnormal lipid biomarker levels and CVD incidence was significant, with increases in CVD risk ranging from 65% for HDL-C levels < 40/50 mg/dL (RR: 1.650, 95% CI: 1.281, 2.124) and 72% for TC levels ≥200 mg/dL (RR: 1.721, 95% CI: 1.351, 2.192) to 117% for TG levels ≥150 mg/dL (RR: 2.173, 95% CI: 1.675, 2.819), 118% for LDL-C levels ≥100 mg/dL (RR: 2.181, 95% CI: 1.482, 3.211) and 165% for Lp(a) levels ≥50 mg/dL (RR: 2.652, 95% CI: 2.012, 4.018).

After adjustment for age, sex, BMI, smoking, MedDietScore, physical activity level, presence of hypertension, hypercholesterolemia and diabetes mellitus at baseline, use of lipid-lowering drugs, and family history of CVD (Table 2), associations between TC, LDL-C and Lp(a) and CVD incidence were attenuated; however, HDL-C and TG still exhibited a significant independent impact on 10-year CVD incidence (RR per 1 mg/dL: 0.983, 95% CI: 0.967, 1.000 and RR per 1 mg/dL: 1.002, 95% CI: 1.001, 1.003, respectively). Among the fully adjusted models, the one incorporating HDL-C showed the highest explanatory ability (R-square = 18.8%), followed by the ones incorporating LDL-C and Lp(a) (R-square = 17.6%), TC (R-square = 17.4%) and TG (R-square = 17.3%). Based on Harrell’s C values, the models with the highest predictive power were those of LDL-C (0.739) and TG (0.707), followed by those of Lp(a) (0.562), HLD-C (0.521) and TC (0.205), while based on cNRI, the model with the best ability of correct reclassification was that of HDL-C (12.6%), meaning that adding HDL-C in the model on top of the other variables correctly reclassified another 13 out of 100 subjects, followed by LDL-C (11.1%), TC (10.4%), Lp(a) (6.7%) and TG (5.0%).

**Table 2:** Associations between blood lipid biomarkers and 10-year CVD incidence in the total study population (*n* = 2020)

	10-year CVD incidence					
	RR	95%CI	***P*** value	Adj. R^**2**^	Harrell’s C	cNRI
TC, mg/dL	1.003	0.999, 1.007	0.111	17.4%	0.205	10.4%
LDL-C, mg/dL	1.002	0.998, 1.007	0.379	17.6%	0.739	11.1%
HDL-C, mg/dL	0.983	0.967, 1.000	0.050	17.8%	0.521	12.6%
TG, mg/dL	1.002	1.001, 1.003	0.012	17.3%	0.707	5.0%
Lp(a), mg/dL	1.003	0.997, 1.010	0.251	17.6%	0.562	6.7%

The results are presented as relative risks (RRs) with their 95% confidence intervals (CIs), as derived from multivariate Cox proportional hazard models

All models were adjusted for age, sex, body mass index, smoking status, MedDietScore, physical activity level, presence of hypercholesterolemia, hypertension and diabetes mellitus at baseline, use of lipid-lowering medication, and family history of cardiovascular disease

*cNRI* Continuous net reclassification improvement, *CVD* Cardiovascular disease, *HDL-C* High-density lipoprotein cholesterol, *LDL-C* Low-density lipoprotein cholesterol; *Lp(a)* Lipoprotein(a), *TC* Total cholesterol, *TG* Triglycerides

Differences in lipid biomarkers between subgroups of the study population according to age, sex, body weight status, diet quality, use of lipid-lowering medication and presence of hypercholesterolemia are presented in Table 3. Older participants (> 45 years) exhibited a more detrimental lipidemic profile characterized by higher levels of TC, LDL-C and TG (all *P* < 0.001) and a trend for higher levels of Lp(a) (*P* = 0.066) than younger participants (≤45 years). Compared to female subjects, males were characterized by higher TC (*P* = 0.011), LDL-C (*P* < 0.001) and TG (*P* < 0.001) and lower HDL-C (*P* < 0.001); however, Lp(a) levels were significantly higher in female participants (*P* = 0.013). Compared to normal-weight subjects, overweight/obese subjects (BMI ≥25 kg/m^2^) exhibited a more detrimental lipidemic profile with higher levels of TC, LDL-C, TG and lower levels of HDL-C (all *P* < 0.001), and a similarly adverse lipidemic pattern was evident in participants with a low level of adherence to the Mediterranean diet compared to those with high adherence (all *P* < 0.001). Participants using lipid-lowering medication also exhibited a worse lipidemic profile [higher TC, LDL-C, TG and Lp(a) and lower HDL-C] than those not on medication, while subjects with hypercholesterolemia (TC ≥200 mg/dL) had higher levels of TC, LDL-C, TG and Lp(a) (all *P* < 0.001) but similar levels of HDL-C compared to normocholesterolemic subjects.

**Table 3:** Blood lipid biomarkers and 10-year CVD incidence according to age, sex, body weight status, adherence to the Mediterranean diet, use of lipid-lowering medication and presence of hypercholesterolemia (*n* = 2020)

	**Age**		***P*** **value**^**a**^
	**≤45 years (*n*** **= 1080)**	**> 45 years (*n*** **= 940)**	
TC, mg/dL	179 (155, 204)	207 (184, 234)	< 0.001
LDL-C, mg/dL	109 (89, 133)	132 (111, 156)	< 0.001
HDL-C, mg/dL	47 (39, 56)	46 (38, 56)	0.121
TG, mg/dL	82 (58, 119)	119 (87, 167)	< 0.001
Lp(a), mg/dL	10.6 (4.4, 23.1)	12.3 (5.5, 25.9)	0.066
10-year CVD incidence, %	5.0	28.0	< 0.001
	**Sex**		
	**Males (*n*** **= 1006)**	**Females (*n*** **= 1014)**	***P*** **value**^**a**^
TC, mg/dL	196 (167, 225)	189 (164, 216)	0.011
LDL-C, mg/dL	124 (102, 149)	116 (93, 143)	< 0.001
HDL-C, mg/dL	41 (35, 50)	52 (44, 60)	< 0.001
TG, mg/dL	115 (80, 168)	85 (61, 121)	< 0.001
Lp(a), mg/dL	10.8 (4.5, 24.6)	12.2 (5.0, 24.7)	0.013
10-year CVD incidence, %	19.7	11.7	< 0.001
	**Body weight status**		***P*** **value**^**a**^
	**Normal-weight (*n*** **= 845)**	**Overweight/obese (*n*** **= 1175)**	
TC, mg/dL	182 (157, 207)	200 (173, 150)	< 0.001
LDL-C, mg/dL	112 (89, 136)	126 (103, 150)	< 0.001
HDL-C, mg/dL	51 (43, 60)	43 (36, 52)	< 0.001
TG, mg/dL	77 (55, 110)	115 (82, 165)	< 0.001
Lp(a), mg/dL	11.2 (4.7, 23.9)	11.5 (4.9, 24.8)	0.823
10-year CVD incidence, %	9.5	20.2	< 0.001
	**Adherence to the Mediterranean diet**		***P*** **value**^**a**^
	**High (*n*** **= 1000)**	**Low (*n*** **= 1020)**	
TC, mg/dL	179 (156, 207)	202 (179, 230)	< 0.001
LDL-C, mg/dL	111 (90, 135)	127 (107, 154)	< 0.001
HDL-C, mg/dL	51 (42, 60)	43 (36, 53)	< 0.001
TG, mg/dL	78 (56, 110)	122 (91, 177)	< 0.001
Lp(a), mg/dL	11.1 (4.8, 24.0)	11.0 (4.5, 24.5)	0.678
10-year CVD incidence, %	6.0	25.3	< 0.001
	**Use of lipid-lowering medication**		***P*** **value**^**a**^
	**Yes (*n*** **= 106)**	**No (*n*** **= 1914)**	
TC, mg/dL	206 (181, 227)	191 (165, 220)	< 0.001
LDL-C, mg/dL	125 (106, 161)	120 (97, 145)	0.027
HDL-C, mg/dL	43 (35, 54)	47 (38, 56)	0.003
TG, mg/dL	136 (99, 200)	97 (67, 142)	< 0.001
Lp(a), mg/dL	15.3 (7.3, 28.4)	11.2 (4.6, 24.5)	0.022
10-year CVD incidence, %	46.6	14.1	< 0.001
	**Presence of hypercholesterolemia**		***P*** **value**^**a**^
	**Yes (*n*** **= 839)**	**No (*n*** **= 1181)**	
TC, mg/dL	227 (105, 147)	170 (151, 185)	< 0.001
LDL-C, mg/dL	151 (136, 170)	102 (86, 117)	< 0.001
HDL-C, mg/dL	46 (39, 57)	47 (39, 57)	0.537
TG, mg/dL	132 (96, 181)	81 (58, 113)	< 0.001
Lp(a), mg/dL	12.6 (5.4, 28.2)	10.1 (4.3, 22.0)	< 0.001
10-year CVD incidence, %	19.8	12.2	< 0.001

Categorical variables are presented as relative frequencies, while numerical variables are presented as medians (1st, 3rd quartile)

^a^ *P* values for between-group comparisons, as derived from Pearson’s chi-square test for categorical variables and the Mann–Whitney U test for numerical variables

*CVD* Cardiovascular disease, *HDL-C* High-density lipoprotein cholesterol, *LDL-C* Low-density lipoprotein cholesterol, *Lp(a)* Lipoprotein(a), *TC* Total cholesterol, *TG* Triglycerides

Multivariate Cox proportional hazard models also revealed a differential impact of lipid biomarkers on 10-year CVD risk according to age, sex, body weight status, quality of diet, use of lipid-lowering medication and presence of hypercholesterolemia (Table 4). Among the blood lipids assessed, TC and LDL-C emerged as significant predictors of CVD risk in ≤45-year-old participants, whereas HDL-C, TG and Lp(a) were associated with CVD risk in older participants (> 45 years). Sex differences were also evident, with TG being associated with CVD risk in males and Lp(a) being associated with CVD risk in females. In normal-weight subjects, TC and LDL-C were significant predictors of 10-year CVD incidence; in contrast, TG was the only blood lipid biomarker with a significant detrimental impact on CVD risk in overweight/obese individuals. Analyses stratified by diet quality revealed that HDL-C and TG were positively associated with CVD risk in participants reporting low adherence to the Mediterranean diet, while none of the blood lipids assessed were significant predictors in those who closely adhered to the Mediterranean diet. Moreover, TC and LDL-C were significant predictors of 10-year CVD incidence in participants not using lipid-lowering medication, while TG was the only lipid associated with CVD risk in those under medication. Finally, associations between blood lipid biomarkers and 10-year CVD incidence were evident only in hypercholesterolemic subjects, in which HDL-C and TG were significant predictors.

**Table 4:** Associations between blood lipid biomarkers and 10-year CVD incidence stratified by age, sex, body weight status, adherence to the Mediterranean diet, use of lipid-lowering medication and presence of hypercholesterolemia (*n* = 2020)

	**10-year CVD incidence**					
	**Age**					
	**≤45 years (*n*** **= 1080)**			**> 45 years (*n*** **= 940)**		
	**RR**	**95%CI**	***P*** **value**	**RR**	**95%CI**	***P*** **value**
TC, mg/dL	1.013	1.001, 1.026	0.030	1.001	0.997, 1.005	0.795
LDL-C, mg/dL	1.013	1.000, 1.027	0.049	0.999	0.994, 1.003	0.516
HDL-C, mg/dL	0.999	0.981, 1.027	0.543	0.990	0.977, 1.000	0.050
TG, mg/dL	1.001	0.997, 1.005	0.692	1.001	1.000, 1.002	0.041
Lp(a), mg/dL	1.003	0.991, 1.016	0.611	1.005	1.000, 1.009	0.048
	**Sex**					
	**Males (*n*** **= 1006)**			**Females (*n*** **= 1014)**		
	**RR**	**95%CI**	***P*** **value**	**RR**	**95%CI**	***P*** **value**
TC, mg/dL	1.002	0.998, 1.007	0.269	1.004	0.997, 1.011	0.301
LDL-C, mg/dL	1.001	0.996, 1.005	0.731	1.004	0.995, 1.013	0.354
HDL-C, mg/dL	0.992	0.976, 1.009	0.347	0.996	0.978, 1.014	0.663
TG, mg/dL	1.001	1.000, 1.002	0.050	1.000	0.997, 1.004	0.937
Lp(a), mg/dL	1.000	0.994, 1.006	> 0.999	1.007	1.001, 1.013	0.024
	**Body weight status**					
	**Normal-weight (*n*** **= 845)**			**Overweight/obese (*n*** **= 1175)**		
	**RR**	**95%CI**	***P*** **value**	**RR**	**95%CI**	***P*** **value**
TC, mg/dL	1.009	1.000, 1.018	0.050	1.002	0.998, 1.006	0.410
LDL-C, mg/dL	1.011	1.000, 1.022	0.048	1.000	0.996, 1.005	0.864
HDL-C, mg/dL	0.992	0.968, 1.017	0.518	0.994	0.980, 1.007	0.370
TG, mg/dL	1.002	0.998, 1.005	0.307	1.001	1.000, 1.002	0.010
Lp(a), mg/dL	1.006	0.998, 1.015	0.153	1.001	0.997, 1.006	0.653
	**Adherence to the Mediterranean diet**					
	**High (*n*** **= 1000)**			**Low (*n*** **= 1020)**		
	**RR**	**95%CI**	***P*** **value**	**RR**	**95%CI**	***P*** **value**
TC, mg/dL	1.003	0.992, 1.014	0.599	1.002	0.998, 1.006	0.304
LDL-C, mg/dL	0.995	0.982, 1.009	0.511	1.001	0.997, 1.005	0.658
HDL-C, mg/dL	0.999	0.963, 1.035	0.943	0.992	0.979, 0.999	0.049
TG, mg/dL	1.008	0.997, 1.019	0.143	1.001	1.000, 1.002	0.050
Lp(a), mg/dL	1.008	0.996, 1.021	0.192	1.002	0.997, 1.007	0.360
	**Use of lipid-lowering medication**					
	**Yes (*n*** **= 106)**			**No (*n*** **= 1914)**		
	**RR**	**95%CI**	***P*** **value**	**RR**	**95%CI**	***P*** **value**
TC, mg/dL	1.000	0.992, 1.006	0.761	1.005	1.000, 1.009	0.031
LDL-C, mg/dL	0.996	0.987, 1.004	0.314	1.004	1.000, 1.009	0.050
HDL-C, mg/dL	0.995	0.959, 1.032	0.780	0.992	0.979, 1.005	0.235
TG, mg/dL	1.003	1.001, 1.006	0.016	1.000	0.999, 1.002	0.461
Lp(a), mg/dL	1.002	0.983, 1.063	0.805	1.003	0.998, 1.008	0.194
	**Presence of hypercholesterolemia**					
	**Yes (*n*** **= 839)**			**No (*n*** **= 1181)**		
	**RR**	**95%CI**	***P*** **value**	**RR**	**95%CI**	***P*** **value**
TC, mg/dL	1.002	0.997, 1.065	0.399	1.003	0.993, 1.012	0.605
LDL-C, mg/dL	1.001	0.996, 1.006	0.768	1.001	0.991, 1.011	0.845
HDL-C, mg/dL	0.980	0.964, 0.996	0.015	1.007	0.990, 1.025	0.424
TG, mg/dL	1.001	1.000, 1.002	0.050	1.000	0.998, 1.002	0.786
Lp(a), mg/dL	1.003	0.997, 1008	0.385	1.003	0.995, 1.011	0.456

The results are presented as relative risks (RRs) with their 95% confidence intervals (CIs), as derived from multivariate Cox proportional hazard models

All models were adjusted for age, sex, body mass index, smoking status, MedDietScore, physical activity level, presence of hypercholesterolemia, hypertension and diabetes mellitus at baseline, use of lipid-lowering medication, and family history of cardiovascular disease, but in each sensitivity model, the variable used for stratification was removed

*CVD* Cardiovascular disease, *HDL-C* High-density lipoprotein cholesterol, *LDL-C* Low-density lipoprotein cholesterol, *Lp(a)* Lipoprotein(a), *TC* Total cholesterol, *TG* Triglycerides

## Discussion

Dyslipidemia has long been established as a core step of the atherosclerosis process and a crucial risk factor for the development of CVD. However, the relationship between a single risk factor and a future CVD event can be significantly modulated by other biological, anthropometric, lifestyle and clinical characteristics. Therefore, the evaluation of the predictive ability of traditional and novel blood lipid biomarkers in different subgroups of the population is important to better understand individualized interactions between the lipidemic profile and health and to improve CVD risk screening systems and prevention strategies.

Herein, using data from a large-scale, prospective, epidemiological study, we demonstrated that several traditional and novel blood lipid biomarkers, i.e., TC, LDL-C, HDL-C, TG and Lp(a), are univariately related to CVD risk; however, associations remain significant only for HDL-C and TG when several potential confounders are taken into account. In addition, the link between blood lipids and CVD risk was found to significantly differentiate in stratified analyses according to other CVD risk factors and participant characteristics. Specifically, TC and LDL-C emerged as significant predictors of CVD risk only among younger subjects, normal-weight subjects, and those not on lipid-lowering medication, while HDL-C and TG emerged as significant predictors of CVD in older subjects, those exhibiting low adherence to the Mediterranean diet, and those with hypercholesterolemia. TG also emerged as a CVD risk-predicting lipid biomarker in male subjects, overweight/obese participants and lipid-lowering medication users, while Lp(a) was found to predict CVD risk in female subjects and older participants. Despite the potential limitations of our study, the present findings shed light on the complex link between blood lipid biomarkers and CVD and could be of value for the design and implementation of more individualized interventions and efficient public health strategies for CVD prevention among specific subgroups of the general population.

In recent decades, the cholesterol/lipid hypothesis, supporting that high levels of TC and LDL-C are the major cause of atherosclerosis and CVD, has dominated the fields of CVD epidemiology, prevention and treatment [28]. As a result, TC and LDL-C have received the most attention in relation to CVD risk, and cholesterol-lowering drugs, most importantly statins, have been widely promoted for CVD prevention. However, evidence supporting the causal link between TC, LDL-C and CVD remains controversial, and recent data have revealed that CVD risk cannot be merely explained by TC and LDL-C levels, that LDL-C might even be beneficial in terms of overall lifespan and that treatment with statins is of doubtful benefit in regard to primary CVD prevention [29, 30]. In the present study, TC and LDL-C were not associated with CVD risk in the total study population of 2020 apparently healthy adult males and females after adjustment for several potential confounders, but their predictive value was significant in specific subgroups, i.e., younger subjects (≤45 years), normal-weight ones, and those not receiving lipid-lowering medication. Our results are in line with previous studies supporting that TC and LDL-C are not the only useful or the most accurate predictors of CVD morbidity and mortality, especially in certain subgroups of the population, such as women, elderly individuals, and normolipidemic individuals not requiring treatment [31–35]. It is possible that the traditional atherogenic lipidemic profile, characterized by elevated TC and LDL-C, is crucial for young healthy individuals and constitutes the initial trigger for CVD onset; however, as atherosclerosis progresses, other lipid biomarkers might become of greater importance. The age-specific link of TC and LDL-C with CVD can also be explained by the mediating role of stress. It has been shown that stress can raise TC and LDL-C due to the necessity of cholesterol for steroid stress hormone production [36, 37], and stress may lead to CVD by promoting inflammation, hypertension and hypercoagulation [38, 39]. This can explain why TC and LDL-C are predictors of CVD risk among younger people who may experience more stress due to increased family or occupational responsibilities compared to older individuals.

Recent evidence suggests that TC and LDL-C only cover a part of the complex interaction between blood lipids and CVD and that other lipid biomarkers might be of equal or even greater importance for CVD risk assessment and prevention [7–9]. The present findings support this concept, since HDL-C and TG were the strongest predictors of CVD, both in the total study cohort and in subgroups of older subjects, those exhibiting poor dietary habits, and those with hypercholesterolemia, while TG also predicted CVD risk in males, overweight/obese participants and lipid-lowering medication users. It is therefore possible that HDL-C and TG are more important blood lipids for older individuals with high cardiometabolic risk. For example, LDL-C has been shown to have a lower predictive value for CVD development among individuals with obesity or metabolic syndrome who present with low HDL-C levels, fasting and postprandial hypertriglyceridemia, elevated small and dense apo B lipoproteins, and thus a high residual CVD risk [4]. In this subgroup of the population, HDL-C and TG might have a better prognostic ability due to the combined effect of metabolic syndrome dyslipidemia, other metabolic components (abdominal fat accumulation, insulin resistance, hypertension, subclinical inflammation and oxidative stress), and the adoption of an unhealthy lifestyle on CVD risk [40, 41]. In this context, the relationship between blood lipid biomarkers and CVD might be significantly influenced by diet quality [42, 43]. For example, the adoption of a prudent/healthy dietary pattern, characterized by a high content of polyunsaturated fatty acids, micronutrients with antioxidant properties and phytochemicals, can beneficially affect blood lipid levels (lowering effect), prevent lipid oxidation, and preserve good endothelial function [44–46]. It is therefore reasonable that the association between blood lipid biomarkers and CVD risk will be attenuated or even absent in subjects with prudent dietary habits, as also supported by our findings showing that none of the blood lipids assessed were significant predictors of CVD risk in participants reporting a high level of adherence to the Mediterranean diet, a dietary pattern rich in health-promoting plant-based foods and numerous phytochemicals with strong antioxidant, anti-inflammatory and cardioprotective properties [47–49].

Among novel lipid biomarkers, Lp(a) has emerged as an independent risk factor for CVD risk and mortality both in the general population and in high-risk individuals, and this association has been found to be continuous even at low LDL-C levels [50–52], a fact that has led to intense research for the development of novel Lp(a)-lowering drugs. According to a 2022 European Atherosclerosis Society consensus statement, “a high Lp(a) concentration should be interpreted in the context of other risk factors and absolute global CVD risk, and addressed through intensified lifestyle and risk factor management” [53]. Interestingly, Lp(a) has been proposed as an emerging lipid biomarker for cardiac health, especially in the female population [54]; this concept is supported by some evidence showing a stronger association between Lp(a) levels and CVD incidence among females than males [55, 56] and improvements in the predictive ability of models assessing global CVD risk in women when Lp(a) is added as a predictor [57], although this female-specific effect has been absent or even contradicted in other studies [58, 59]. Our results highlight the detrimental impact of high Lp(a) levels on CVD risk among older subjects and females and thus support age- and sex-related differences in the impact of Lp(a) on CVD risk. In this context, Lp(a) levels have been shown to be closely associated with female sex hormones and to increase after menopause, a fact that might partly explain the significant postmenopausal increase in CVD risk and the cardioprotective effect of hormone replacement therapy in women after menopause [60, 61]. Given that we were not able to perform additional subgroup sensitivity analyses (e.g., according to both age and sex) due to the relatively small number of CVD events recorded at 10 years, the potential value of Lp(a) as a predictor of CVD among specific subgroups of the population, such as postmenopausal women not on estrogen therapy, remains to be explored in future studies.

### Study strengths and limitations

The present findings add to the limited knowledge on the prospective association between blood lipids and CVD incidence in relation to other important CVD risk factors and characteristics, including age, sex, body weight, diet quality, use of lipid-lowering medication and presence of hypercholesterolemia, and provide evidence of a complex and unique independent interaction between blood lipids and CVD risk in different subgroups of the general population. The adequate study sample, the prospective design of the study, the long follow-up period, and the comprehensive assessment of participants’ characteristics at baseline are strengths of this work.

However, the present study is also characterized by several limitations. The main drawback is that only baseline measurements of lipid biomarkers were available; therefore, the prospective dynamic change in blood lipids in relation to 10-year CVD incidence could not be explored. Moreover, since we examined associations between blood lipid biomarkers and CVD incidence in apparently healthy subjects with low CVD risk, we cannot conclude the role of TC, LDL-C, HDL-C, TG and Lp(a) in subjects with high CVD risk or patients with established CVD. In addition, LDL-C was calculated through the Friedewald formula and not directly measured via ultracentrifugation, which is the gold standard method for LDL-C estimation; although widely adopted in clinical practice for several decades, the Friedewald equation is prone to inaccuracy at low LDL-C and/or high TG levels, where errors in estimating very-low-density lipoprotein cholesterol are magnified due to the use of a fixed factor to describe its relationship with TG [62]. Additional limitations of the present study include the fact that dietary habits were self-reported by participants and the data obtained can be prone to recall bias, although the food frequency questionnaire used was validated for the Greek population and completed with the assistance of trained dietitians, and the study sample consisted of Caucasian subjects living in the Attica region (78% urban municipalities), which does not allow the generalization of the present findings in the whole Greek population (e.g., subjects living in rural areas) or populations of a different ethnic background.

## Conclusions

While dyslipidemia has long been established as a risk factor for CVD, a more detailed and individualized research approach is required to identify specific lipid biomarkers that can predict CVD risk in different population groups and design efficient interventions toward CVD prevention. The present study aimed to shed light on this understudied topic and supports that the link between blood lipids and CVD differentiates according to several biological, lifestyle and clinical parameters. Specifically, TC and LDL-C, traditionally used to assess CVD risk and guide therapeutic decisions, were associated with CVD incidence only among younger subjects, normal-weight subjects, and those not on lipid-lowering medication. In contrast, HDL-C and TG emerged as significant predictors of CVD among higher-risk subgroups, including older subjects, overweight/obese subjects, those with less compliance with the principles of the Mediterranean diet, and those with hypercholesterolemia. Sex differences were also evident, with TG and Lp(a) differentially predicting CVD risk in males and females, respectively. Further large-scale, multicenter prospective epidemiological studies are needed to confirm the generalization of the presented findings and contribute to the elucidation of the role of traditional and novel blood lipid biomarkers in CVD screening and prevention.

## Data Availability

The data that support the findings of this manuscript were used under license and are not yet publicly available. Data are, however, available from the authors upon reasonable request and with permission of the ATTICA study.

## References

[1] Pirillo A, Casula M, Olmastroni E, Norata GD, Catapano AL (2021). Global epidemiology of dyslipidaemias. Nat Rev Cardiol.

[2] Conroy RM, Pyorala K, Fitzgerald AP, Sans S, Menotti A, De Backer G (2003). Estimation of ten-year risk of fatal cardiovascular disease in Europe: the SCORE project. Eur Heart J.

[3] Ference BA, Ginsberg HN, Graham I, Ray KK, Packard CJ, Bruckert E (2017). Low-density lipoproteins cause atherosclerotic cardiovascular disease. 1. Evidence from genetic, epidemiologic, and clinical studies. A consensus statement from the European atherosclerosis society consensus panel. Eur Heart J.

[4] Sampson UK, Fazio S, Linton MF (2012). Residual cardiovascular risk despite optimal LDL cholesterol reduction with statins: the evidence, etiology, and therapeutic challenges. Curr Atheroscler Rep.

[5] Wong ND, Zhao Y, Quek RGW, Blumenthal RS, Budoff MJ, Cushman M (2017). Residual atherosclerotic cardiovascular disease risk in statin-treated adults: the multi-ethnic study of atherosclerosis. J Clin Lipidol.

[6] Lieb W, Enserro DM, Larson MG, Vasan RS (2018). Residual cardiovascular risk in individuals on lipid-lowering treatment: quantifying absolute and relative risk in the community. Open Heart.

[7] Drexel H, Aczel S, Marte T, Vonbank A, Saely CH (2010). Factors predicting cardiovascular events in statin-treated diabetic and non-diabetic patients with coronary atherosclerosis. Atherosclerosis.

[8] Nomikos T, Panagiotakos D, Georgousopoulou E, Metaxa V, Chrysohoou C, Skoumas I (2015). Hierarchical modelling of blood lipids’ profile and 10-year (2002-2012) all cause mortality and incidence of cardiovascular disease: the ATTICA study. Lipids Health Dis.

[9] Orozco-Beltran D, Gil-Guillen VF, Redon J, Martin-Moreno JM, Pallares-Carratala V, Navarro-Perez J (2017). Lipid profile, cardiovascular disease and mortality in a Mediterranean high-risk population: the ESCARVAL-RISK study. PLoS One.

[10] Nordestgaard BG, Chapman MJ, Ray K, Borén J, Andreotti F, Watts GF (2010). Lipoprotein(a) as a cardiovascular risk factor: current status. Eur Heart J.

[11] Malaguarnera M, Vacante M, Russo C, Malaguarnera G, Antic T, Malaguarnera L (2013). Lipoprotein(a) in cardiovascular diseases. Biomed Res Int.

[12] Matsuura Y, Kanter JE, Bornfeldt KE (2019). Highlighting residual atherosclerotic cardiovascular disease risk. Arterioscler Thromb Vasc Biol.

[13] Mosca L, Barrett-Connor E, Wenger NK (2011). Sex/gender differences in cardiovascular disease prevention: what a difference a decade makes. Circulation.

[14] Andersson C, Vasan RS (2018). Epidemiology of cardiovascular disease in young individuals. Nat Rev Cardiol.

[15] Casas R, Castro-Barquero S, Estruch R, Sacanella E. Nutrition and cardiovascular health. Int J Mol Sci. 2018;19(12):3988. 10.3390/ijms19123988 PMC6320919 30544955

[16] Powell-Wiley TM, Poirier P, Burke LE, Despres JP, Gordon-Larsen P, Lavie CJ (2021). Obesity and cardiovascular disease: a scientific statement from the American Heart Association. Circulation.

[17] Sofi F, Macchi C, Abbate R, Gensini GF, Casini A (2014). Mediterranean diet and health status: an updated meta-analysis and a proposal for a literature-based adherence score. Public Health Nutr.

[18] Tosti V, Bertozzi B, Fontana L (2018). Health benefits of the Mediterranean diet: metabolic and molecular mechanisms. J Gerontol A Biol Sci Med Sci.

[19] Pitsavos C, Panagiotakos DB, Chrysohoou C, Stefanadis C (2003). Epidemiology of cardiovascular risk factors in Greece: aims, design and baseline characteristics of the ATTICA study. BMC Public Health.

[20] World Medical Association Declaration of Helsinki (2000). Ethical principles for medical research involving human subjects. JAMA.

[21] Friedewald WT, Levy RI, Fredrickson DS (1972). Estimation of the concentration of low-density lipoprotein cholesterol in plasma, without use of the preparative ultracentrifuge. Clin Chem.

[22] Mach F, Baigent C, Catapano AL, Koskinas KC, Casula M, Badimon L (2020). 2019 ESC/EAS guidelines for the management of dyslipidaemias: lipid modification to reduce cardiovascular risk. Eur Heart J.

[23] Kushner RF (2012). Clinical assessment and management of adult obesity. Circulation.

[24] Alberti KG, Eckel RH, Grundy SM, Zimmet PZ, Cleeman JI, Donato KA (2009). Harmonizing the metabolic syndrome: a joint interim statement of the international diabetes federation task force on epidemiology and prevention; National Heart, Lung, and Blood Institute; American Heart Association; world heart federation; international atherosclerosis society; and International Association for the Study of obesity. Circulation.

[25] Papathanasiou G, Georgoudis G, Papandreou M, Spyropoulos P, Georgakopoulos D, Kalfakakou V (2009). Reliability measures of the short international physical activity questionnaire (IPAQ) in Greek young adults. Hell J Cardiol.

[26] Katsouyanni K, Rimm EB, Gnardellis C, Trichopoulos D, Polychronopoulos E, Trichopoulou A (1997). Reproducibility and relative validity of an extensive semi-quantitative food frequency questionnaire using dietary records and biochemical markers among Greek schoolteachers. Int J Epidemiol.

[27] Panagiotakos DB, Pitsavos C, Stefanadis C (2006). Dietary patterns: a Mediterranean diet score and its relation to clinical and biological markers of cardiovascular disease risk. Nutr Metab Cardiovasc Dis.

[28] Brown WV (1990). Review of clinical trials: proving the lipid hypothesis. Eur Heart J.

[29] Ravnskov U, de Lorgeril M, Diamond DM, Hama R, Hamazaki T, Hammarskjöld B (2018). LDL-C does not cause cardiovascular disease: a comprehensive review of the current literature. Expert Rev Clin Pharmacol.

[30] Diamond DM, Ravnskov U (2015). How statistical deception created the appearance that statins are safe and effective in primary and secondary prevention of cardiovascular disease. Expert Rev Clin Pharmacol.

[31] Ulmer H, Kelleher C, Diem G, Concin H (2004). Why eve is not Adam: prospective follow-up in 149650 women and men of cholesterol and other risk factors related to cardiovascular and all-cause mortality. J Women's Health (Larchmt).

[32] Ravnskov U (2002). Is atherosclerosis caused by high cholesterol?. QJM.

[33] Ravnskov U, Diamond DM, Hama R, Hamazaki T, Hammarskjold B, Hynes N (2016). Lack of an association or an inverse association between low-density-lipoprotein cholesterol and mortality in the elderly: a systematic review. BMJ Open.

[34] Bathum L, Depont Christensen R, Engers Pedersen L, Lyngsie Pedersen P, Larsen J, Nexoe J (2013). Association of lipoprotein levels with mortality in subjects aged 50 + without previous diabetes or cardiovascular disease: a population-based register study. Scand J Prim Health Care.

[35] Hamazaki T, Okuyama H, Ogushi Y, Hama R (2015). Towards a paradigm shift in cholesterol treatment. A re-examination of the cholesterol issue in Japan. Ann Nutr Metab.

[36] Dimsdale JE, Herd JA (1982). Variability of plasma lipids in response to emotional arousal. Psychosom Med.

[37] Rosenman RH. Relationships of neurogenic and psychological factors to the regulation and variability of serum lipids. 1993;9:133–40.

[38] Steptoe A, Kivimäki M (2012). Stress and cardiovascular disease. Nat Rev Cardiol.

[39] Dar T, Radfar A, Abohashem S, Pitman RK, Tawakol A, Osborne MT (2019). Psychosocial stress and cardiovascular disease. Curr Treat Options Cardiovasc Med.

[40] Ruotolo G, Howard BV (2002). Dyslipidemia of the metabolic syndrome. Curr Cardiol Rep.

[41] Paraskevas KI, Karatzas G, Pantopoulou A, Iliopoulos DG, Perrea D (2010). Targeting dyslipidemia in the metabolic syndrome: an update. Curr Vasc Pharmacol.

[42] Denke MA (1995). Cholesterol-lowering diets. A review of the evidence. Arch Intern Med.

[43] Clifton PM (2019). Diet, exercise and weight loss and dyslipidaemia. Pathology..

[44] Georgoulis M, Yiannakouris N, Tenta R, Fragopoulou E, Kechribari I, Lamprou K (2021). A weight-loss Mediterranean diet/lifestyle intervention ameliorates inflammation and oxidative stress in patients with obstructive sleep apnea: results of the “MIMOSA” randomized clinical trial. Eur J Nutr.

[45] Georgoulis M, Yiannakouris N, Kechribari I, Lamprou K, Perraki E, Vagiakis E, et al. Cardiometabolic benefits of a weight-loss Mediterranean diet/lifestyle intervention in patients with obstructive sleep apnea: the “MIMOSA” randomized clinical trial. Nutrients. 2020;12(6):1570. 10.3390/nu12061570 PMC7352432 32481487

[46] Scicchitano P, Cameli M, Maiello M, Modesti PA, Muiesan ML, Novo S (2014). Nutraceuticals and dyslipidaemia: beyond the common therapeutics. J Funct Foods.

[47] Georgoulis M, Georgousopoulou EN, Chrysohoou C, Pitsavos C, Panagiotakos DB. Longitudinal trends, determinants, and Cardiometabolic impact of adherence to the Mediterranean diet among Greek adults. Foods. 2022:11(16):2389. 10.3390/foods11162389 PMC9407264 36010387

[48] Georgoulis M, Kontogianni MD, Yiannakouris N (2014). Mediterranean diet and diabetes: prevention and treatment. Nutrients.

[49] Schwingshackl L, Morze J, Hoffmann G (2020). Mediterranean diet and health status: active ingredients and pharmacological mechanisms. Br J Pharmacol.

[50] Forbes CA, Quek RGW, Deshpande S, Worthy G, Wolff R, Stirk L (2016). The relationship between Lp(a) and CVD outcomes: a systematic review. Lipids Health Dis.

[51] Wilson DP, Jacobson TA, Jones PH, Koschinsky ML, McNeal CJ, Nordestgaard BG (2019). Use of lipoprotein(a) in clinical practice: a biomarker whose time has come. A scientific statement from the National Lipid Association. J Clin Lipidol.

[52] Fogacci F, Cicero AFG, D'Addato S, D'Agostini L, Rosticci M, Giovannini M (2017). Serum lipoprotein(a) level as long-term predictor of cardiovascular mortality in a large sample of subjects in primary cardiovascular prevention: data from the Brisighella heart study. Eur J Int Med.

[53] Kronenberg F, Mora S, Stroes ESG, Ference BA, Arsenault BJ, Berglund L, et al. Lipoprotein(a) in atherosclerotic cardiovascular disease and aortic stenosis: a European atherosclerosis society consensus statement. Eur Heart J. 2022;43(39):925-3946. 10.1093/eurheartj/ehac361 PMC9639807 36036785

[54] Manson JE, Bassuk SS (2015). Biomarkers of cardiovascular disease risk in women. Metabolism.

[55] Frohlich J, Dobiasova M, Adler L, Francis M (2004). Gender differences in plasma levels of lipoprotein (a) in patients with angiographically proven coronary artery disease. Physiol Res.

[56] Markus MRP, Ittermann T, Schipf S, Bahls M, Nauck M, Volzke H (2021). Association of sex-specific differences in lipoprotein(a) concentrations with cardiovascular mortality in individuals with type 2 diabetes mellitus. Cardiovasc Diabetol.

[57] Ridker PM, Buring JE, Rifai N, Cook NR (2007). Development and validation of improved algorithms for the assessment of global cardiovascular risk in women: the Reynolds risk score. JAMA.

[58] Kouvari M, Panagiotakos DB, Chrysohoou C, Georgousopoulou EN, Yannakoulia M, Tousoulis D (2019). Lipoprotein (a) and 10-year cardiovascular disease incidence in apparently healthy individuals: a sex-based sensitivity analysis from ATTICA cohort study. Angiology.

[59] Cook NR, Mora S, Ridker PM (2018). Lipoprotein(a) and cardiovascular risk prediction among women. J Am Coll Cardiol.

[60] Kim CJ, Ryu WS, Kwak JW, Park CT, Ryoo UH (1996). Changes in Lp(a) lipoprotein and lipid levels after cessation of female sex hormone production and estrogen replacement therapy. Arch Intern Med.

[61] Taskinen MR, Puolakka J, Pyorala T, Luotola H, Bjaorn M, Kaarianen J (1996). Hormone replacement therapy lowers plasma Lp(a) concentrations. Comparison of cyclic transdermal and continuous estrogen-progestin regimens. Arterioscler Thromb Vasc Biol.

[62] Maki KC, Grant JK, Orringer CE (2022). LDL-C estimation: the perils of living with imperfection. J Am Coll Cardiol.

